# Fam-Trastuzumab-Deruxtecan and Osimertinib Combination to Target HER2 Driven Resistance in a Patient With NSCLC After Osimertinib Progression: Case Report

**DOI:** 10.1016/j.jtocrr.2025.100787

**Published:** 2025-01-07

**Authors:** Hailey M. Hirata, Carrie B. Lee, Kevin Y. Chen

**Affiliations:** a Department of Pharmacy, University of North Carolina Hospitals, Chapel Hill, North Carolina; b Lineberger Comprehensive Cancer Center, University of North Carolina at Chapel Hill, Chapel Hill, North Carolina; c Department of Medicine, University of North Carolina at Chapel Hill, Chapel Hill, North Carolina

**Keywords:** Osimertinib, Fam-trastuzumab-deruxtecan, EGFR-positive NSCLC, HER2 targeted treatment, Osimertinib resistance, Case report

## Abstract

HER2 mutations have been identified as potential mechanisms of resistance to EGFR-directed therapies in patients with advanced or metastatic NSCLC. Here, we report the case of a 65-year-old female with metastatic, EGFR exon 19–mutant NSCLC initially treated with first-line osimertinib. After several subsequent lines of treatment including erlotinib and carboplatin+pemetrexed+osimertinib, repeat genetic sequencing identified a HER2 exon 20 insertion (A775_G776insYVMA) in both blood and tissue. She was treated using fam-trastuzumab-deruxtecan, which resulted in a disease response lasting for 8 months. This report represents the first published case detailing the safety and efficacy of a combination fam-trastuzumab-deruxtecan and osimertinib in a patient with NSCLC and HER2-mutated resistance after EGFR-targeted therapy. The findings from this report suggest that fam-trastuzumab-deruxtecan can be safely given in combination with osimertinib and should be considered as subsequent-line therapy for patients who progress on osimertinib and develop a HER2 resistance mutation.

## Introduction

After the development of molecular testing, targeted therapy against identified driver mutations has become a cornerstone of treatment in NSCLC. Recently, HER2 mutations have been identified as potential therapeutic targets, with fam-trastuzumab-deruxtecan exhibiting promising efficacy and receiving the approval of the U.S. Food and Drug Administration for both HER2-mutant and HER2-amplified NSCLC. However, there remains limited data detailing the use of HER2-directed therapies after osimertinib progression. To our knowledge, this case represents the first published report detailing the use of a combination osimertinib and fam-trastuzumab-deruxtecan for the treatment of EGFR and HER2-mutated NSCLC after progression on osimertinib.

## Case Report

**Figure 1:**
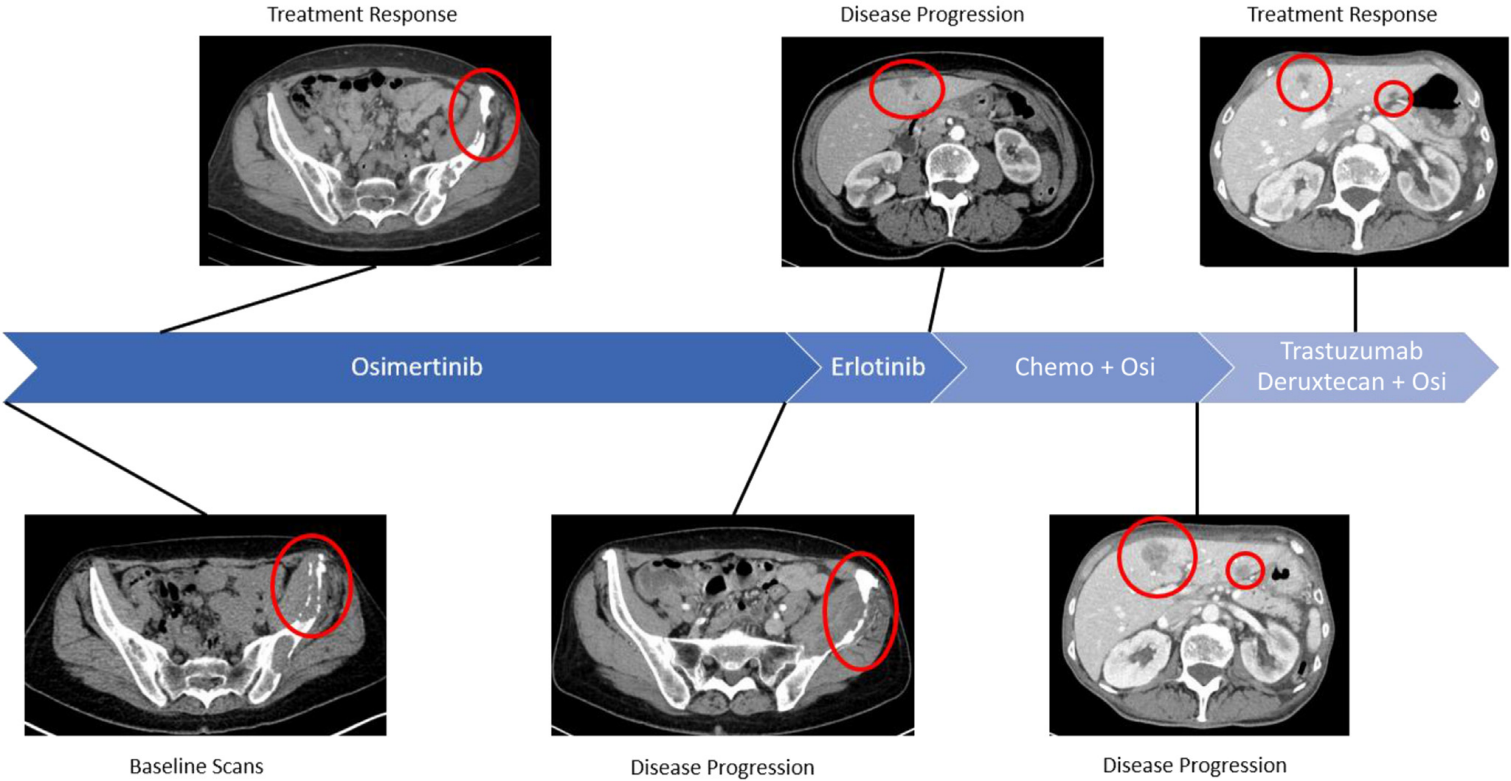
Treatment history and therapy response. Chemo, chemotherapy; Osi, osimetrinib.

**Figure 2:**
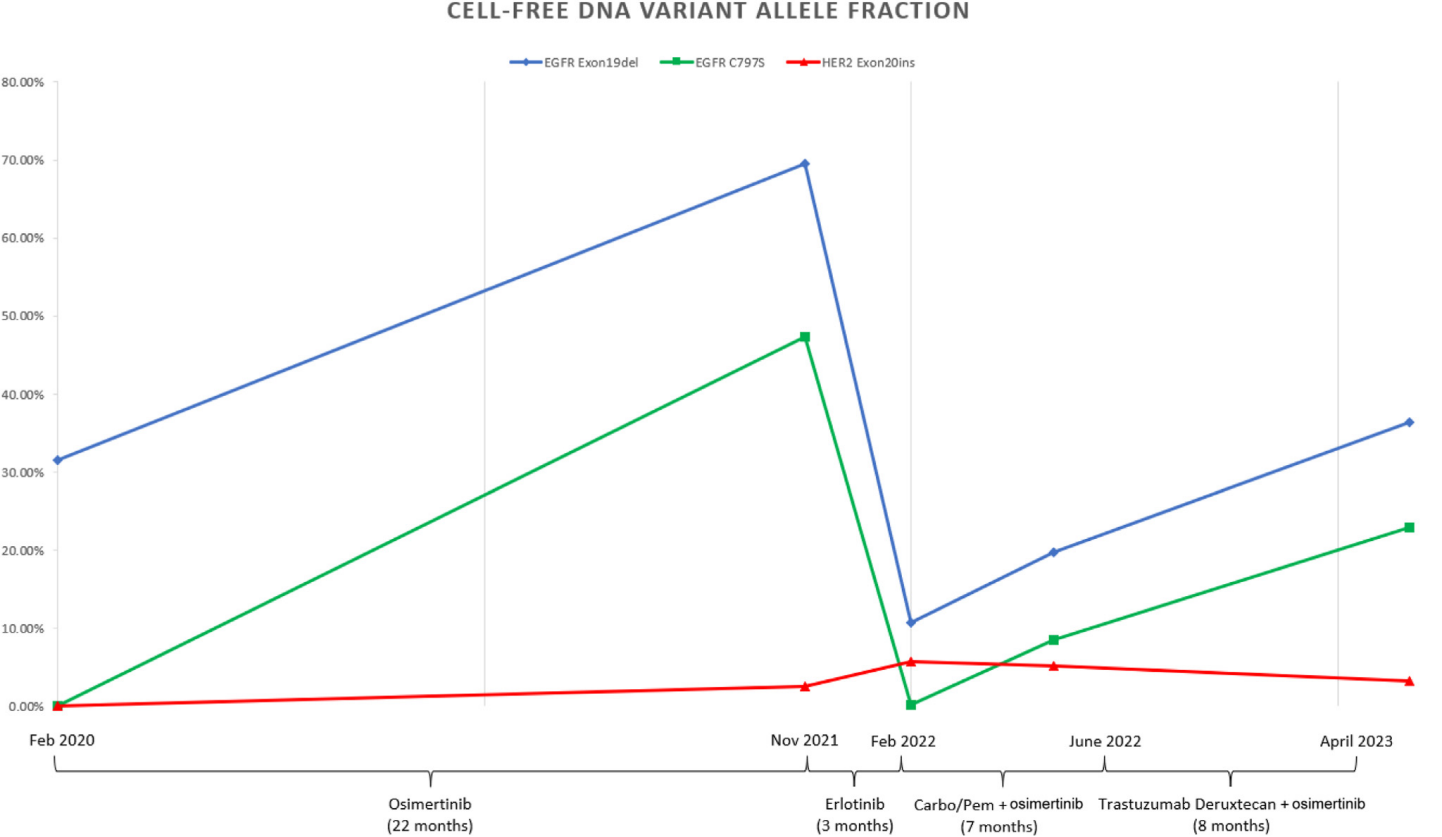
Select somatic mutations detected by cfDNA. CfDNA, cell-free DNA; exon19 del, Exon 19 deletion; Exon20 ins, exon 20 insertion; Feb, February; Nov, November; Osi, osimetrinib.

Our patient is a 65-year-old female originally diagnosed with metastatic, EGFR exon 19–mutated lung adenocarcinoma in January 2020. Comprehensive molecular profiling with cell-free DNA (cfDNA) identified the following mutations: EGFR exon 19 (E746_A750del), BRAF amplification, and CCNE1 amplification. A HER2 mutation was not detected on her baseline molecular testing. She was initially treated with osimertinib 80 mg daily with stable disease for 22 months. In November of 2021, computed tomography (CT) scan revealed multiple osseous lesions (new right first rib pathologic fracture, left iliac wing [Fig. 1]) and enlarging lymph nodes (psoas, external iliac). The patient had worsening pain in the left iliac wing, which confirmed both clinical and radiographic disease progression. Repeat molecular using cfDNA revealed a new EGFR C797S resistance mutation with the highest variant allele frequency (VAF) of 47.4% (Fig. 2), with additional new alteration of HER2 exon 20 (A775_G776insYVMA; VAF: 2.6%). Osimertinib was subsequently discontinued, and she was switched to single-agent erlotinib treatment given the C797S mutation. The patient remained on therapy for 4 months before developing progression in the liver and brain (Fig. 1). Repeat cfDNA molecular testing revealed a reduction of EGFR C797S subclone (VAF: 0.2%) with no new alterations. Notably, the HER2 exon 20 insertion mutation remained detectable (VAF: 5.8%) at this time. Erlotinib was discontinued and she was restarted on osimertinib given central nervous system progression, and systemic chemotherapy with carboplatin and pemetrexed. After four cycles of carboplatin, pemetrexed and osimertinib, the patient was transitioned to maintenance pemetrexed plus osimertinib. CT scans revealed stable disease for approximately 5 months, at which time there was a clear progression in the liver (Fig. 1). A liver biopsy was performed, which revealed adenocarcinoma with the original EGFR (E746_A750del) and the same HER2 mutation (A775_G776insYVMA) seen on cfDNA, confirming the resistant liver subclones were HER2-driven. Given these findings, she started a combination treatment with fam-trastuzumab-deruxtecan and osimertinib. Fam-trastuzumab-deruxtecan was initiated at a dose of 5.4 mg/kg every 21 days and osimertinib was continued at a dose of 80 mg daily, and restaging scans after two and four cycles revealed ongoing treatment response in the liver lesions (Fig. 1). The patient experienced considerable fatigue, nausea, and neutropenia necessitating several dose reductions of fam-trastuzumab-deruxtecan. She tolerated a dose of 4.4 mg/kg for the longest duration of time (four cycles). Ultimately, owing to fatigue and nausea, she elected to take a break after eight cycles of treatment, continuing on with osimertinib monotherapy. After a month's break from fam-trastuzumab-deruxtecan, restaging scans revealed progressive disease in the liver and brain. Repeat cfDNA testing revealed no new driver mutations and increased EGFR C797S VAF. Because of decreased performance status, the patient was not felt to be a candidate for further fam-trastuzumab-deruxtecan or other systemic therapies and was transitioned to hospice.

## Discussion

Treatment after osimertinib progression remains a therapeutic challenge as there is a paucity of data to guide a standard approach. Resistance to osimertinib occurs by three potential mechanisms: EGFR-dependent, EGFR-independent, or histologic transformation.^1^ Our strategy for this patient exemplifies the common practice of obtaining repeat tissue biopsy and molecular sequencing to identify potential mechanisms of osimertinib resistance, then using targeted therapy approaches to overcome this resistance. Notably, a key challenge is tumor heterogeneity, which may necessitate multiple treatment approaches. Although this patient’s initial osseous progression on osimertinib was driven by a C797S resistance mutation, the switch to second-line erlotinib resulted in rapid loss of central nervous system disease control. Subsequently, the progression in the liver was mediated by a HER2 exon 20 insertion mutation, necessitating a HER2-directed therapy. When selecting off-label single agent or combination therapy, tolerability should be considered as there may be a risk of overlapping toxicities such as diarrhea, fatigue, nausea, myelosuppression, and pneumonitis. As such, it is imperative to have a balanced discussion with the patient or seek regulatory approval per local guidelines before starting treatment.

Evidence has emerged revealing the benefit of using resistance-guided approaches. In a study conducted by Choudhury et al.,^2^ 29% (n = 95) of patients treated with first-line osimertinib underwent postprogression biopsies and repeat molecular testing to detect resistance mechanisms. Of these patients, 53% (n = 50) had an identified mechanism of resistance including on-target, off-target, or histologic transformation. Though limited in sample size, the authors found that patients receiving tailored therapy for resistance had a significant 24-month improvement in survival compared with those who did not (hazard ratio = 0.09, *p* = 0.006).^2^ Similarly, the INSIGHT 2 trial revealed the benefit of adding tepotinib in patients with acquired MET amplifications after first-line osimertinib.^3^ In their cohort, an overall response was observed in 50% of patients with a median duration of response of 8.5 months in patients treated with the osimertinib and tepotinib combination. The prospective ORCHARD trial is underway to identify the benefit of subsequent targeted therapies.

Literature also exists for the use of HER2-directed therapy in patients with acquired mutations after osimertinib treatment. Notably, two of the 26 patients in the resistance-directed therapy group in the previous study received ado-trastuzumab emtansine.^2^ The prospective phase 1-2 Trastuzumab-Emtansine and Osimertinib trial was conducted evaluating the combination of T-DM1 and osimertinib in patients with acquired HER2 overexpression.^4^ In their 27-patient cohort, overall response was seen in 4% of patients with a median progression-free survival of 2.8 months. Although these results are discouraging, the limited efficacy may possibly be because of the poor predictive ability of HER2 overexpression for HER2-directed therapy in NSCLC. Recent findings from the DESTINY-Lung01 trial using fam-trastuzumab-deruxtecan suggest HER2 mutations (primarily in exon 20), may serve as a better biomarker of efficacy.^5^

## Conclusions

To our knowledge, this case report is the first to report outcomes from the combined use of fam-trastuzumab-deruxtecan and osimertinib for the treatment of EGFR and HER2-mutant NSCLC after progression on osimertinib therapy. On the basis of this patient, who experienced approximately 8 months of clinical benefit, the addition of fam-trastuzumab-deruxtecan to osimertinib may be a safe and effective treatment option in patients who develop HER2 resistance mutations after osimertinib progression.

## CRediT Authorship Contribution Statement

**Hailey M. Hirata:** Conceptualization, Writing - original draft.

**Carrie B. Lee:** Writing - reviewing and editing.

**Kevin Y. Chen:** Conceptualization, Writing - original draft, Writing - reviewing and editing.

## Disclosure

The authors declare no conflict of interest.
